# Transcriptional Profiling of a Yeast Colony Provides New Insight into the Heterogeneity of Multicellular Fungal Communities

**DOI:** 10.1371/journal.pone.0046243

**Published:** 2012-09-28

**Authors:** Ana Traven, Amrei Jänicke, Paul Harrison, Angavai Swaminathan, Torsten Seemann, Traude H. Beilharz

**Affiliations:** 1 Department of Biochemistry & Molecular Biology, Monash University, Clayton, Victoria, Australia; 2 Victorian Bioinformatics Consortium, Monash University, Clayton, Victoria, Australia; New Jersey Medical School, University of Medicine and Dentistry of New Jersey, United States of America

## Abstract

Understanding multicellular fungal structures is important for designing better strategies against human fungal pathogens. For example, the ability to form multicellular biofilms is a key virulence property of the yeast *Candida albicans*. *C. albicans* biofilms form on indwelling medical devices and are drug resistant, causing serious infections in hospital settings. Multicellular fungal communities are heterogeneous, consisting of cells experiencing different environments. Heterogeneity is likely important for the phenotypic characteristics of communities, yet it is poorly understood. Here we used colonies of the yeast *Saccharomyces cerevisiae* as a model fungal multicellular structure. We fractionated the outside colony layers from the cells in the center by FACS, using a Cit1-GFP marker expressed exclusively on the outside. Transcriptomics analysis of the two subpopulations revealed that the outside colony layers are actively growing by fermentative metabolism, while the cells residing on the inside are in a resting state and experience changes to mitochondrial activity. Our data shows several parallels with *C. albicans* biofilms providing insight into the contributions of heterogeneity to biofilm phenotypes. Hallmarks of *C. albicans* biofilms – the expression of ribosome and translation functions and activation of glycolysis and ergosterol biosynthesis occur on the outside of colonies, while expression of genes associates with sulfur assimilation is observed in the colony center. Cell wall restructuring occurs in biofilms, and cell wall functions are enriched in both fractions: the outside cells display enrichment of cell wall biosynthesis enzymes and cell wall proteins, while the inside cells express cell wall degrading enzymes. Our study also suggests that noncoding transcription and posttranscriptional mRNA regulation play important roles during growth of yeast in colonies, setting the scene for investigating these pathways in the development of multicellular fungal communities.

## Introduction

Unicellular yeasts can associate into multicellular structures such as colonies, flocs, flors, stalks, mats and biofilms [1], [2]. Understanding multicellular behaviors of fungi is important for combating human disease caused by fungal pathogens, such as *Candida albicans*. *C. albicans* forms biofilms on indwelling medical devices, and these structures are resistant to antifungal treatments [1], [2], [3]. This makes biofilm-related infections very difficult to treat, resulting in high mortality rates [2], [3].

The development of multicellular fungal communities is controlled by complex differentiation pathways [2]. Importantly, the cells growing in the multicellular community differ substantially from their unicellular counterparts. For example, cells from flocs of *S. cerevisiae* (which form by cells adhering to each other via the action of cell wall adhesins) are more resistant to several forms of stress than non-flocculent cells [4]. Similarly, C. *albicans* biofilm-derived cells are more resistant to antifungal drugs than their planktonic counterparts [5]. Moreover, transcriptome profiling of *S. cerevisiae* colonies and biofilms of *Candida* species revealed that cells growing as a multicellular community display gene expression profiles distinct from single cells growing in liquid media, with one of the main features being metabolic reprogramming [6], [7], [8], [9], [10], [11], [12].

Multicellular fungal communities are structurally complex. The cells in the community have different access to nutrients and oxygen, and overall experience different “neighborhoods”. This means that the cells in the community are heterogeneous, and likely perform distinct physiological roles. An example of such heterogeneity is the presence of a small number of persister cells in *C. albicans* biofilms, which are more resistant to antifungal drugs than the rest of the biofilm or planktonic cultures [13]. Dissecting the features of the individual subpopulations and their contributions to the phenotypes of the community has the potential to provide new insight into targeting fungal biofilms with therapeutic agents.

Colonies formed by the bakers yeast *S. cerevisiae* on agar plates have served as a model for understanding multicellular behaviors of yeasts [6], [10]. In previous work, protein-GFP fusions or promoter-lacZ fusions have been used to identify genes that are differentially expressed in the different parts of the colony (the outside versus the inside cell layers) [10], [14]. Using these studies as the basis, we surveyed several strains from the yeast GFP collection [15] to identify a protein-GFP fusion that would allow us to separate cells from the outside layers of the colony from those on the colony inside by FACS-based sorting. Transcriptome analysis of the two subpopulations from yeast colonies revealed substantial metabolic reprogramming within the colony. The transcriptome of a *S. cerevisiae* colony resembles in multiple aspects that of a *Candida* biofilm, and the differential regulation of gene expression within a yeast colony provides insight into the contributions of cell heterogeneity to colony and biofilm phenotypes.

## Materials and Methods

### Yeast Strains and Growth Conditions

BY4741 (*MATa his3*Δ*0 leu2*Δ*0 met15*Δ*0 ura3*Δ*0*) was utilized as the genetic background to generate the Ato1-GFP strain by PCR mediated epitope tagging (ATO1-GFP For GGCTCGTCCATTCCCATTACCATCTACTGAAAGGGTAATCTTTcgtacgctgcaggtcgac; CCR4-GFP Rev CAGAGAGGAGGGAGGGAGTGGGATGAAAGTGTGCGGTttaatcgatgaattcgagctcg) [16]. The Ccr4-, Ade5,7-an d Cit1-GFP fusions were derived from the Howson (2005) collection and were a gift from Antony Cooper (Garvan Institute; Australia). All cells were grown in YPAD media (1% yeast extract, 2% Peptone, 0.004% Adenine hemisulfate and 2% glucose) either in liquid culture or on agar plates grown at 30°C. Cells grown on solid media were diluted from overnight cultures and plated with glass beads to achieve a final colony density of ∼300 distinct colonies per 10 cm petri dish.

### Microscopy

Day-4 colonies were stabilized by layering with 1% low-melt agarose to ∼1 mm above the top of the colony. The colony was then sliced in half and the cut-side placed facing down on a coverslip essentially as previously described [10]. The GFP fluorescence was captured using a Leica SP5 inverted microscope with the 10× objective. Leica LAS-AF software was used for analysis.

### FACS Analysis and Sorting

**Figure 1:**
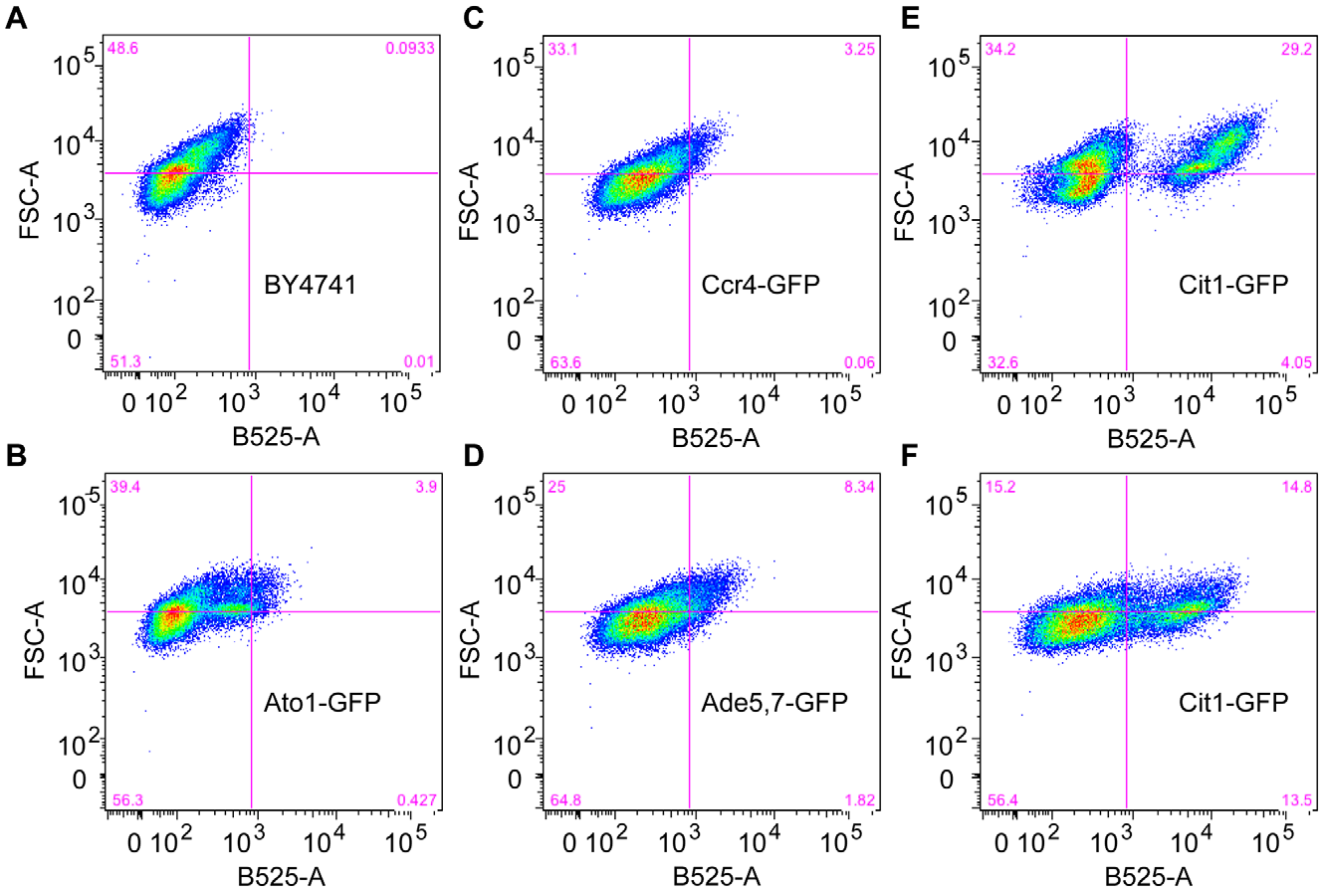
FACS analysis of yeast strains expressing protein-GFP fusions in stationary phase and colony growth. Shown are FACS plots. The y-axis represents forward scatter (FSC-A), where an increased signal can indicate increased cell size or budding. The x-axis indicates GFP fluorescence (B525-A). Pink sight lines are included to guide the eye to size and fluorescence differences between the wild-type strain (A) and GFP- fusion stains (B–F). Day-4 yeast cells from the indicated strains were harvested either by washing from the surface of YPAD agar plates (A–D and F), or from stationary phase liquid cultures (E).

Day-4 colonies were washed from the surface of YPAD-plates with ∼5 ml ice-cold dH_2_O and stored on ice. For analysis (Figure 1) 30,000 events were captured using a BD Biosciences LSRII flow cytometer. Flow Jo software was used in the analysis. To sort the GFP populations a minimum of 1 million GFP +ve and GFP-ve cells were captured using a BD Biosciences Influx cell sorter. The gates for sorting were based on the natural separation of the distinct GFP +ve population in the Cit1-GFP strain. The gate for the GFP-ve population was based on the population coordinates of the untagged strain. This meant that dimly fluorescent cells (∼25%) within the population are excluded from the analysis. This was performed with 3 biological replicates temporally separated by over one week.

### Transcriptome Analysis

Arrays were designed using eArray (Agilent Technologies). We included probes based on transcribed coordinates supplied in supplemental data by Xu et al (2009). This set contains coding and non-coding (CUTs and SUTs) and structural RNA such as snoRNAs. We also included the “YBOX” probe set containing a number of control and normalization probes designed by Pat Brown’s lab at Stanford. Finally, the generic *S. cerevisiae* probes provided by manufacturer were also included. Each probe was spotted at least twice and provided more than one suitable probe could be designed, multiple probes were designed to each transcript (60,000 spotted probes in total). The array design is freely available via the eArray website (Design ID: 031069, Array Design: Beilharz Sc_01). Microarray processing was performed by The Ramaciotti Centre for Gene Function Analysis (http://www.ramaciotti.unsw.edu.au) using the low-input kit (Agilent Technologies).

Three biological repeats and one dye-swap were analysed in total. The average log2 intensity of the negative control probes was 4.83, standard deviation 0.32. A log2 average intensity cut off of 5.47 (two standard deviations above mean negative control intensity) was applied. Moderated t-tests were used to look for differential expression, using the limma package in BioConductor, and a FDR cutoff of 0.01. Where multiple probes spanned a single transcribed region the data were averaged.

### RNA Extraction and Analysis

RNA was isolated from yeast cells using the hot phenol method, and the concentration estimated using a nanodrop spectrophotometer. For poly(A)tail analysis the ePAT and TVN-PAT assays were performed exactly as previously described [17] using a total of 500 ng of RNA as input into each cDNA synthesis reaction. The primer sequences used were the following: S.c ATO1-PAT CTTATATAACCACACCAACTAATCG; S.c ENO2-PAT CCACCACGGTGACAAGTTG; S.c HSP30-PAT GCAGAGCAAGCTGTCGAAG; S.c SNR6-PAT GTTCGCGAAGTAACCCTTCG; S.c HPF1-PAT GAATGCCAATACTTTGAATGCATTG; S. c SUT350-PAT GCCACAACACAGGGCCTAG. Note: Unlike most PAT primers, the SUT350 primer is designed near the start of the annotated SUT350 transcript.

## Results

### Establishing Methods for FACS Sorting of the Colony Subpopulations

**Figure 2:**
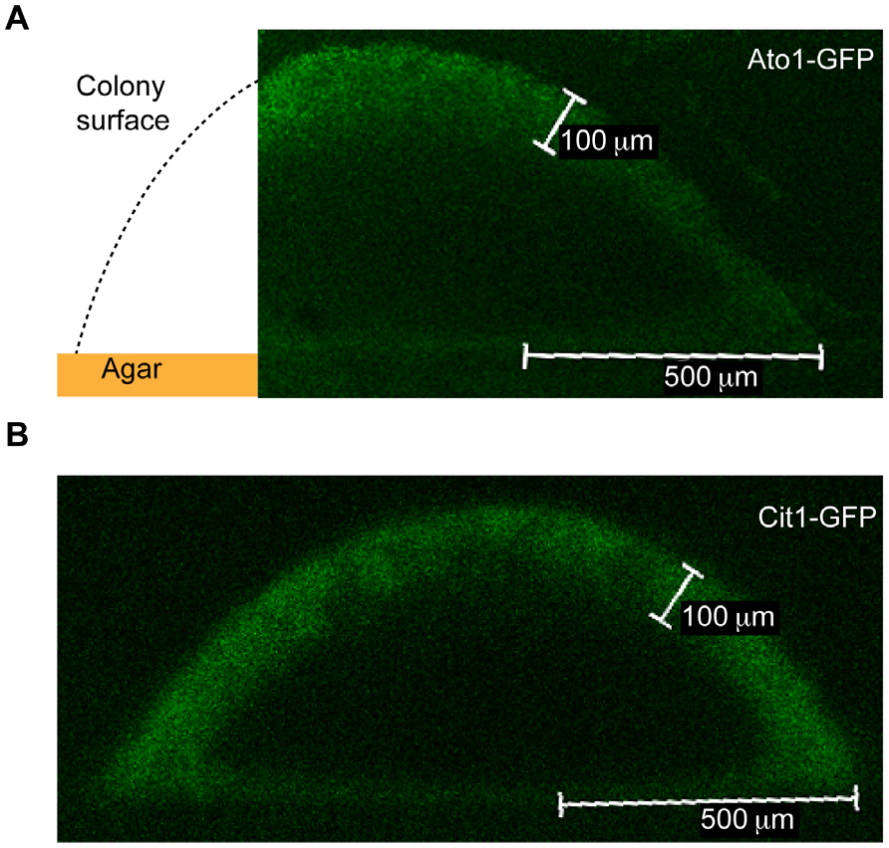
Cit1-GFP is expressed in the outside layer of cells in a colony. Confocal laser microscopy of colony cross-sections from either Ato1-GFP (A), or Cit1-GFP strains (B) shows a band of GFP-fluorescent cells ∼100 microns thick at the surface of the colony (10× objective). In the Ato1-GFP micrograph, the colony shape and agar surface are extended in schematic for orientation (A).

Previously, a GFP-fusion protein has been used to demonstrate that the ammonium exporter Ato1 is differentially expressed within the colony in a spatial manner, with expression evident in the outer layer of the yeast colony, but absent on the inside [10]. Ammonia production is thought to be a central metabolic feature of yeast colonies that drives their development [6], [18]. The expression of Ato1-GFP in the outside colony layers coincides with a fully differentiated colony, which is in ammonia-producing phase [10]. The Palkova group has also previously reported the localization of *CCR4* and *ADE 5,7*- LacZ reporter fusions as being predominantly on the inside of colonies [14]. We performed FACS analysis of the untagged BY4741 wild-type strain, and Ato1-GFP, Ccr4-GFP and Ade5,7-GFP strains after 4 days of colony growth on rich media (Figure 1A–D). The Ato1 fusion revealed a small separate GFP positive population of cells (note cells shifted in the B525-A axis) that tended to correspond to larger cells (up shifted in FSC-A axis). Neither Ccr4-GFP nor Ade5,7-GFP expressing colonies revealed clear subpopulations. In these strains the GFP fluorescence was dim and GFP positive cells tended to be the biggest cells in the population. This suggests, contrary to previous findings, that these genes are most abundantly expressed on the colony exterior (see cells shifted to the upper right hand quadrant of the FACS plots relative to the untagged strain). We also tested another gene, *CIT1*, which is a marker of quiescent cells in stationary phase cultures [19]. This gene was chosen because cells growing in a colony display several features of quiescence. FACS analysis of Cit1-GFP cells from stationary phase liquid cultures confirmed the distinct quiescent subpopulation previously observed by Davidson et al 2011 (Figure 1E). Moreover, Cit1-GFP cells grown in colonies also demonstrated the presence of two distinct cell populations (Figure 1F). The GFP positive population accounted for approximately 25% of the cells, a further ∼25% was dimly fluorescent, while the remaining 50% of cells shared FACS co-ordinates with the untagged wild-type control strain. Again, the fluorescent cells tended to have a higher signal in forwards scatter (y-axis) indicating bigger or budding cells. Confocal fluorescence microscopy showed that Cit1-GFP is indeed expressed on the outside of differentiated yeast colonies, in a pattern analogous to that previously shown for Ato1-GFP (Figure 2, and [10]). Since the Cit1-GFP strain produced the most distinctly separable populations by FACS, we decided to use this strain to sort the two subpopulations of the colony, distinguishing the outside cell layers from those in the inside.

### Transcriptome Analysis of the Two Subpopulations within a Yeast Colony

The outside and inside cells from a colony were sorted based on GFP-fluorescence, total RNA was isolated and transcriptome analysis performed using custom 2-colour agilent arrays containing multiple probes for both coding and non-coding RNA. Our arrays were designed to contain probes for the complete set of transcribed coordinates identified by tiling arrays [20] (see Methods). The gene expression of the GFP positive cells was directly compared to the GFP negative cells by competitive hybridization. Genes with elevated expression in each of the colony subpopulations were identified relative to total gene expression in that particular subpopulation, using 1.5 fold enrichment and a false discovery rate of 1% (see Materials and methods). The complete normalised microarray dataset is presented in Dataset S1.

### Genes Enriched on the Outside of the Colony

The expression of three hundred and twelve genes was enriched on the outside of the colony. A prominent functional group was genes required for ribosome biogenesis and translation (including 53 genes encoding ribosomal subunits) (Table 1; Gene ontology analysis is shown in Table 2). Prominent metabolic functions included glycolysis and glucose fermentation, ergosterol biosynthesis and fatty acid metabolism, metabolism of amino acids and vitamins, as well as enzymes required for acetyl-coA synthesis and lactate utilization (Tables 1 and 2). Expression of ribosome biogenesis genes and the enzymes of glycolysis and glucose fermentation pathway indicate that the outside of the colony contains actively growing cells that metabolise glucose in the preferred way for yeast fermentation. However, enrichment of genes of the fatty acid oxidation pathway, acetyl-coA synthesis and amino acid metabolism indicates that, while the cells on the outside of the colony are metabolically active, they also exhibiting some properties of starved cells.

**Table 1:** List of selected functional categories, genes and non-coding transcripts enriched on the outside of the colony.

FUNCTION	GENES
**Ribosome biogenesis and translation**	53 genes encoding subunits of the cytoplasmic ribosome; ribosome and rRNA biogenesis factors (ECM1, ECM16, EGD2, LOC1, MAK21, RIA1, RIX1, RRP1, RRP3, URB1, URB2, UTP8, UTP10, UTP21); translation factors and tRNA synthetases (EFT1, EFT2, SES1, CDC60, DED1, FRS1, RBG2, GCN20, ACS1)
**Enzymes of glycolysis and gluconeogenesis, glucose fermentation pathway**	PGK1, ENO1, ENO2, TDH2, TDH3, FBA1, TPI1, PDC1, ADH1, ADH2, ALD4
**Fatty acid oxidation and biogenesis**	Fatty acid oxidation enzymes (POT1, POX1, FOX2, FAA2, FAA4, DCI1, ECI1); other factors involved in the beta-oxidation pathway (TES1, ANT1, CTA1, PCD1); fatty acid biosynthesis enzymes (FAS1, ELO1); ACB1 (Acyl-CoA binding protein; transports newly synthesized acyl-CoA esters to acyl-CoA-consuming processes)
**Ergosterol biosynthesis**	Ergosterol biosynthesis enzymes (ERG1, ERG5, ERG6, ERG11, ERG25, ERG13; ERG10); other factors required for ergosterol biosynthesis and transport (ERG28, DET1)
**Metabolism of amino acids and nucleotides**	Amino acid catabolism (ARO10, ARO9. ICL2); amino acid biosynthesis (ARO4, HIS7, ILV3, ARO9, MDE1, MRI1, AAT1, ACO1); pentose phosphate pathway (TKL2); purine and pyrimidine biosynthesis (ADE6, ADK1, URA2, URA7, RNR2, RNR4); transporters (TAT1, ALP1)
**Biosynthesis of vitamins and NAD**	Biotin biosynthesis (BIO2, BIO4); folate biosynthesis (FOL3, MIS1, GCV2); riboflavin biosynthesis (RIB2); NAD biosynthesis enzymes (NMA1, NPT1); nicotinic acid permease TNA1
**Mitochondria**	Respiratory chain and ATP synthase (CYB2, NCA2, NCA3, STF1, SUE1); putative mitochondrial proteins (YDL157C, FMP37, YER077C); heme biosynthesis (HEM15); mtDNA maintenance (CLU1, MGM101, RIM1); Other (SHH3, MCX1, CIR1, DIC1)
**Other metabolism**	Acetyl-coA synthesis (ACS1, ACS2); coenyzme A synthesis (CAB1); TCA cycle (ACO1, LSC2); utilization of lactate (DLD2)
**Cell wall**	Cell wall proteins (FIT3, TIR1, PIR1, PIR3, CIS3, HSP150, PST1, TIP1,YLR194C, CWP2, CCW14, EMW1, AGA2); cell wall biogenesis: glucan and chitin (FKS1, CHS2, KNH1); mannosyltransferase (PMT4)
**Cell cycle, DNA replication and repair**	Cell cycle and polarized growth (CDC10, RGA2, CLA4, GIC2, CLN1, NKP1, APC5, SMC2, SWI6, MSA2); DNA replication and repair (RNR2, RNR4, POL2, POL4, POL31, POL30, DNA2, SMC5, RFC1, DMC1, ECL1, CIN1, ABF1)
**Transcription**	Mediator subunit SIN4/MED16 required for stationary phase survival; SAGA subunit SPT7 (mutant has stress responsive and cell cycle phenotypes); TFIID subunit TAF2; Cell cycle (SWI6, MSA2, ABF1)
**Stress response**	Nutrient stress, starvation and stationary phase (ATO2, ATO3, SNZ2, TOS3, SIN4); Oxidative stress (SOD2, TSA1, YPL108W, ALO1, OLA1, STF2); Osmotic stress (GPD1, STL1, GRE1, SIP18, PPZ1, YWC1); Heat shock proteins and general stress response (HSP26, SSA3, HMF1, UBC5)
**ER and Golgi**	Protein glycoslylation (WBP1); ER and nuclear pore complex association (PER33); Protein folding in the ER (EMC4); Vesicle trafficking (ERV29, SED4, ERP1); Protein targeting to the ER (SRP68, SEC63, SEC65), Other (FPR2, IRC22); Golgi (COY1, COG5, VPS54, GGA2)
**Noncoding transcripts (SUTs and CUTs)**	CUT406, CUT410, CUT525, CUT757, CUT866, SUT058, SUT121, SUT530, SUT565, SUT660, SUT664, SUT761

The complete list of genes enriched on the outside of the colony is shown in Dataset S1. CUT-cryptic unstable transcript; SUT-stable unannotated transcript.

**Table 2:** Gene ontology analysis of differential gene expression within the colony.

Functions enriched on the outside of the colony	
GO category	p value
structural constituent of ribosome [GO:0003735]	4.41E-11
translation [GO:0006412]	7.12E-11
rRNA export from nucleus [GO:0006407]	1.12E-05
maturation of SSU-rRNA from tricistronic rRNA transcript (SSU-rRNA, 5.8S rRNA, LSU-rRNA) [GO:0000462]	3.02E-05
snRNP protein import into nucleus [GO:0006608]	7.1E-03
snRNA export from nucleus [GO:0006408]	7.09E-03
ergosterol biosynthetic process [GO:0006696]	4.08E-05
fatty acid metabolic process [GO:0006631]	1.1E-04
gluconeogenesis [GO:0006094]	1.9E-04
reactive oxygen species metabolic process [GO:0072593]	4E-04
lactate metabolic process [GO:0006089]	6.5E-03
acetyl-CoA biosynthetic process [GO:0006085]	2.2E-03
alcohol fermentation [MIPS functional classification 02.16.01]	0.2E-03
C-2 compound and organic acid catabolism [MIPS functional classification 01.05.06.07]	0.5E-03
propionate fermentation [MIPS functional classification 02.16.11]	2.2E-03
metabolism of derivatives of dehydroquinic acid, shikimic acid and chorismic acid [MIPS functional classification 01.20.15]	4.9E-03
structural constituent of cell wall [GO:0005199]	4.76E-07
peroxisomal matrix [GO:0005782]	7.1E-03
**Functions enriched on the inside of the colony**	
**GO category**	
electron carrier activity [GO:0009055]	4.3E-03
respiratory electron transport chain [GO:0022904]	1.05E-3
proton-transporting ATP synthase complex, coupling factor F(o) [GO:0045263]	9.3E-03
mitochondrial electron transport, cytochrome c to oxygen [GO:0006123]	7.5E-03
ammonium transmembrane transport [GO:0072488]	3.31E-05
detoxification of cadmium and copper ion [GO:0071585; 0010273]	1.03E-3
cytokinesis, completion of separation [GO:0007109]	1.4E-03
negative regulation of gluconeogenesis [GO:0045721]	2.4E-03
diacetyl reductase ((R)-acetoin forming) activity [GO:0052587]	1.03E-03
cellular response to water deprivation [GO:0042631]	9.7E-03
response to pH [GO:0009268]	9.7E-03
nitrogen utilization [GO:0019740]	2.4E-03
general transcription activities [MIPS classification 11.02.03.01]	7.3E-03
degradation of polyamines [MIPS classification 01.01.05.01.02]	5.9E-03
cation transport (H+, Na+, K+, Ca2+, NH4+, etc.) [MIPS classification 20.01.01.01]	0.3E-03
cAMP/cGMP mediated signal transduction [30.01.09.07]	5.9E-03
inorganic chemical agent resistance (e.g. heavy metals) [32.05.01.03.03]	1.05E-03

Only minimally overlapping GO terms are shown. The full GO analysis is presented in Dataset S1.

In addition to metabolism and ribosome biogenesis, several cell wall genes were enriched on the outside of the colony, in particular genes encoding cell wall proteins, and enzymes required for cell wall synthesis, such as ß 1,3 glucan synthase *FKS1*, chitin synthase *CHT2*, and *KNH1* that is required for the synthesis of ß 1,6 glucans (Table 1). Several of the cell wall protein genes are known to be regulated by stress responses, such as heat and cold shock, mitochondrial dysfunction, anaerobiosis, and the cell wall integrity pathway (*TIR1*, *TIP1*, *HSP150*, *PIR1*, *PIR3*, *PST1*, *YLR194C*). These results are consistent with the outside colony layer exhibiting properties of starved and quiescent cells, which are known to remodel their cell walls. Other functional groups enriched on the outside of the colony include transcription factors, such as the *MED16* subunit of Mediator known to be required for stationary phase survival [21], genes involved in cell cycle, polarized growth and DNA replication and repair, and several stress responsive functions, which include the *ATO2* and *ATO3* ammonium transporters that have long been known to be induced during colony development [6].

Grouping of the genes enriched on the outside of the colony by transcription factors using YEASTRACT (www.yeastract.com) and selecting for “direct evidence” (i.e. transcription factors likely to have a direct effect) found several transcription factors with potential roles in regulating the colony transcriptome. We will list those with more prominent roles (regulation of >10% of the genes in the list). 50.6% of the genes were regulated by Ste12, a master regulator of pseudohyphal growth in response to nitrogen starvation. Interestingly, the *ste12* mutant displays altered colony morphology [22]. Together, the study by Granek and Magwene (2010) and our results indicate that Ste12 might be an important colony regulator. As expected, other prominent transcription factors were those involved in ribosome biogenesis (Rap1 36.9% of genes, Fhl1 29.8% of genes, Ifh1 19.2% of genes, Sfp1 15.7% of genes). Moreover, 17.3% of the genes enriched on the outside of the colony are under the control of Sok2, a known regulator of ammonia production and differentiation in yeast colonies [23]. Several stress-responsive transcription factors, such as Skn7(14.4% of the genes), Sko1 (12.5% of the genes) and Yap6 (11.9% of the genes) were also identified, as were the pseudohyphal regulators Tec1 (11.5% of the genes) and Phd1 (13.5% of genes), and the iron-sensing transcription factor Yap5 (12.2% of the genes).

### Genes Enriched in the Inside of the Colony

Two hundred and thirteen genes displayed enriched expression on the inside of the colony. The transcriptome profile of the inside of the colony was very different to that of the cells residing on the outside (Table 3, GO analysis is shown in Table 2). Most notably, there was no enrichment in ribosome biogenesis or glycoloysis, consistent with the inside cells having less capacity for growth. Instead, the expression of the key enzyme of gluconeongenesis *PCK1* was enriched. However, somewhat counter intuitively, so were three negative regulators of gluconeogenesis: *UBC8*, *GID8* and *VID28*, which are involved in proteasome-dependent degradation of the gluconeogenesis enzyme fructose-1, 6-bisphosphatase in response to switching from a non-fermentable carbon source to glucose [24], [25]. Several regulators of glucose-dependent pathways were enriched in the colony center, but again, both gluconeogenesis activators (such as the transcription factor *CAT8*) and transcription factors that mediate glucose repression such as *MIG1* and *NRG1*, were identified (Table 3) [26]. The inside cells displayed features of nitrogen starvation, as indicated by an enrichment in the expression of several genes under the control nitrogen catabolite repression (e.g. ammonium permeases *MEP2* and *MEP3*, the allantoate permease *DAL5*, the allantoicase *DAL2* and the proline oxidase *PUT1*), as well as genes required for biosynthesis and transport of amino acids (Table 3).

**Table 3:** List of selected functional categories, genes and noncoding transcripts enriched on the inside of the colony.

FUNCTION	GENES
**Mitochondria**	Respiratory chain subunits encoded on the mtDNA (ATP6, ATP8, COB1, OLI1, COX2, COX3); CYC7 (cytochrome C expressed under hypoxic conditions); cytochrome C oxidase assembly factors (PET100, COA2; COX19); DLD3 (D-lactate dehydrogenase activated by mitochondrial dysfunction); transcriptional regulators of mitochondrial biogenesis and respiratory growth (HAP4, RSF1); other (APJ1)
**Fe-S clusters and iron**	Fe-S clusters biogenesis and assembly (SSQ1, ISU2, NBP35, DRE2); iron transporters (FTR1, FET4); transcription factors responsive to iron (CTH1, CAD1, MSN1)
**Amino acid metabolism, Nitrogen starvation**	Transporters, uptake (MEP2, MEP3, MUP3, DAL5, LST8, AVT6); STP1 (transcription factor, activator of amino acid permease genes); AQR1 (plasma membrane transporters required for excretion of excess amino acids); metabolic enzymes required for amino acid biosynthesis and utilization of alternative nitrogen sources (SER3, TMT1, DAL2, MET14, MET2, CPA1, PUT1, SAM2)
**Glucose-regulated pathways**	Transcription factors (MIG1, NRG1, HAP4, CAT8, IMP2’); signal transduction: cAMP-PKA pathway (PDE1, PDE2); negative regulators of glucose signaling (MTH1, RGS2); SKS1 (putative kinase, adaptation to low glucose); CSR2 (proposed to regulate utilization of non-fermentable carbon sources)
**Gluconeogenesis**	Enzymes (PCK1); negative regulators of gluconeogenesis (UBC8, GID8, VID24)
**Lipid metabolism and regulation**	Sphingolipids (YPC1, SUR1, SUR2, YNL194C); phospholipids (OPI3, PAH1, CLD1, FPK1, PLB3, INO4); fatty acids (FAA1)
**Butanediol biosynthesis (fermentation of pyruvate)**	BDH1, BDH2
**Ion homeostasis and transport**	Detoxification (CUP1-1, CUP1-2, BSD2); transport (PHO89, ENA1, SMF2, SAT4); transcription factor ZAP1 (responsive to zinc); HEF3 (translation factor expressed in zinc deficient cells)
**Cell wall**	Cell wall proteins (SPI1, PRY3); cell wall modifying enzymes: glucanses (EGT2, DSE2, DSE4), chitinase (CTS1); regulators (KIC1 kinase required for cell wall integrity, ZEO1 membrane protein, regulates the PKC-dependent cell wall stress pathway).
**Stress response**	Nutrient deprivation, stationary phase (HSP30, MOH1, RBA50, SSA4, YJL144W); other stresses (GRX6, YDL012C, HSP42, AHA1, CA1, SSA4, YER130C, SKN7, ROX3, SPI1, CMK2, ZEO1, MSN1, YGK3)
**Noncoding transcripts (CUTs, SUTs and snoRNAs)**	20 SNR genes (snoRNAs); RPR1 (RNA component of nuclear RNaseP); SCR1 (RNA component of Signal Recognition Particle); CUTs (CUT419, CUT420, CUT428, CUT438, CUT439, CUT600, CUT643, CUT672, CUT673, CUT734, CUT807, CUT843, CUT917, CUT918); SUTs (SUT024, SUT032, SUT098, SUT102, SUT161, SUT174, SUT178, SUT178, SUT185, SUT243, SUT285, SUT308, SUT326, SUT329, SUT350, SUT409, SUT650)

The complete list of differentially expressed genes is shown in Dataset S1. CUT-cryptic unstable transcript; SUT-stable un-annotated transcript; snoRNA-small nucleolar RNA.

A striking feature of the cells that reside in the inside of the colony was an enrichment of genes (both nuclear and mitochondrially-encoded) required for the activity of the mitochondrial respiratory chain, as well as the expression of the transcription factor *HAP4* (a subunit of the HAP2/3/4/5 complex that is a central regulator of genes required for respiration), and genes required for the biogenesis and assembly of iron-sulfur (Fe-S) clusters, iron transporters and transcription factors responsive to iron (Tables 3 and 2). These changes indicate that the cells residing in the inside of the colony are attempting to maintain mitochondrial activity even in the absence of growth. The cells on the inside of the colony also displayed changes to genes with functions in cell wall metabolism, however this was notably different from the changes observed in the outside cell layers. The inside cells did not show an enrichment in the expression of genes encoding cell wall proteins (with the exception of two genes, one of which *SPI1* is induced at the diauxic shit in planktonic cultures) [27]). Moreover, the inside cells did not express cell wall biosynthesis enzymes (such as glucan and chitin synthases, which were enriched on the outside of the colony), but rather they expressed cell wall degrading enzymes, such as glucanases (*EGT2*, *DSE2* and *DSE4*) and the chitinase *CTS1* (Table 3). We speculate that this provides a mechanism to mobilize carbohydrate stored in the cell wall.

Analysis of the transcription factors for which direct evidence exist for regulation of the genes enriched in the inside of the colony using YEASTRACT showed a very similar picture as for the outside cells. Again, only the transcription factors with control over >10% of the genes will be mentioned. A large proportion of genes (49.3%) are known to be regulated by Ste12, the colony regulator Sok2 exhibits control over 27.1% of the genes, and the stress responsive factors Skn7, Cin5, Sko1, Yap1, Yap6 and Hsf1 control 15.8%, 13.8%, 11.8%, 11.3%, 10.8% and 10.3% of genes respectively. Also identified were the generalist transcription factors Rap1 and Fhl1 (34.5% and 21.7% of genes respectively), the pseudohyphal regulators Phd1 (16.7% of the genes) and Tec1 (13.8% of the genes), and the cell cycle regulators Swi4 (10.8% of the genes).

### Noncoding Transcription in a Yeast Colony

**Figure 3:**
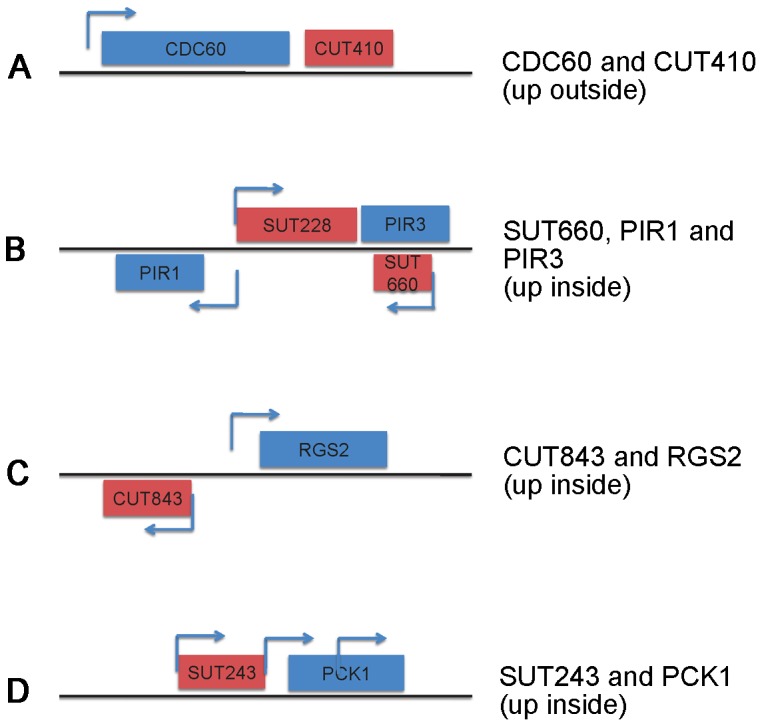
Noncoding transcription in yeast colonies. The CUTs (cryptic unstable transcripts) and SUTs (stable unannotated transcripts) were mapped to the genome using the tools at http://steinmetzlab.embl.de/NFRsharing/ [20]. Examples of the location of noncoding transcripts and their neighboring genes are shown (the drawings are not to scale), and their expression in the outside or inside of the colony is indicated.

Our transcriptome analysis identified several noncoding transcripts that were enriched either on the inside, or on the outside of the colony (Tables 1 and 3). These include cryptic unstable transcripts (CUTs), stable un-annotated transcripts (SUTs), small nucleolar RNA genes (snoRNA, SNR genes), the RNA component of RNaseP and the RNA component of the Signal Recognition Particle. Many more noncoding transcripts were enriched of the inside than on the outside of the colony (53 versus 12). All of the snoRNA genes were enriched on the inside [20]. In regards to the CUTs and SUTs, 31 were enriched in the cells that reside on the inside, versus 12 on the outside. The snoRNAs are required for rRNA biogenesis. Ribosome biogenesis was strongly induced on the outside of the colony, while, counter intuitively, the snoRNAs were enriched on the inside (Tables 1 and 3). As part of the decay mechanism, the snoRNAs are adenylated [28], which would make them more prone to be captured by reverse transcription. We suggest that the apparent enrichment of the snoRNAs on the inside of the colony might actually be due to increased activity of the decay pathway on this set of genes in the absence of active ribosome biogenesis. The CUTs and SUTs were mapped onto the genome to identify the neighboring genes, using the Steinmetz lab web-site (http://steinmetzlab.embl.de/NFRsharing/) that accompanies the manuscript by Xu et al (2009). In 16 cases the expression of the neighboring genes was enriched in the same subpopulation of cells as the noncoding transcript. Some examples are shown in Figure 3. Several noncoding transcripts could be originating from bidirectional promoters, which drive expression of a gene that is also enriched (for example CUT843 and *RGS2* in Figure 3). In some cases, the non-coding transcript was placed upstream of the gene that was also enriched in expression (such as SUT243 and *PCK1*, Figure 3), suggesting that active transcription of the noncoding transcript could be contributing to the expression of the downstream gene. In other cases the opposite was true, i.e. the noncoding transcript was downstream of a highly expressed gene, suggesting its higher levels might be a byproduct of active transcription of the upstream gene (*CDC60* and CUT410 in Figure 3). Several examples included noncoding transcripts overlapping the expressed genes transcribed in the opposite direction on the other strand. There were also more complex situations, such as the case of *PIR1* and *PIR3* (two cell wall genes enriched on the outside) and SUT660 (a noncoding transcript enriched on the outside), in which SUT660 is upstream of *PIR1* transcribed in the same direction, but antisense to *PIR3*. Another such complex example includes SUT329, the ammonium permease *MEP2* and CUT807 that were all enriched in the inside of the colony (see Table 3). SUT329 is upstream of *MEP2*, while CUT807 is antisense (Figure 3). These results collectively suggest that noncoding transcription could be contributing to the shaping of the transcriptome of a yeast colony in a complex manner.

### Posttranscriptional mRNA Regulation in the Developmental Differentiation of a Yeast Colony

**Figure 4:**
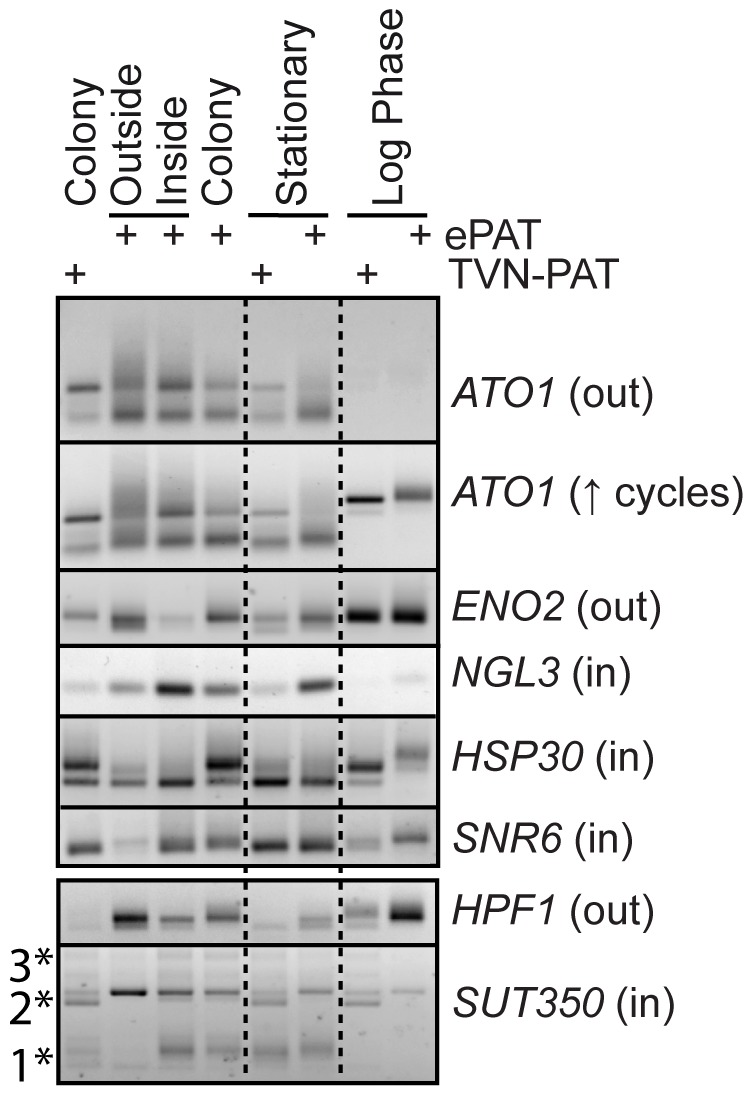
Posttranscriptional mRNA regulation in the yeast colony. Semi-quantitative ePAT and TVN-PAT reactions were utilized to confirm the expression in the outside or inside colony layers, and to identify differences in 3′UTR dynamics. Included are cDNAs generated from the colony subpopulations, the complete colony, day-4 stationary phase liquid cultures and log phase cultures. The ePAT reaction includes the full poly(A)-tail (seen as a smear of PCR amplicons), whereas the TVN-PAT reaction is anchored to the adenylation site with an invariant A-12 poly(A)-tail (usually a tight band). Multiple bands of different sizes indicates alternate polyadenylation site usage. The site of enrichment for each tested mRNA in the arrays is indicated in brackets (in) or (out) after the gene name. All PCRs were 28 cycles, except *ATO1* (↑ cycles) panel, where the PCR cycle number was increased to 30. The * indicates alternate transcriptional termination in the SUT350 transcript.

In addition to transcriptional regulation, posttranscriptional mechanism of mRNA control, such as control of poly(A) tail length, stability, translation rates and alternative 3′ UTR usage, contribute significantly to gene expression [29], [30], [31], [32]. Previous work which used promoter-lacZ fusions suggested that the major cytoplasmic mRNA deadenylase Ccr4 is more highly expressed on the inside than on the outside of the colony [14]. Our transcriptome analysis did not find changes in *CCR4* gene expression (Dataset S1), and using a Ccr4-GFP fusion we did not observe clear subpopulations of GFP-expressing and non-expressing cells, unlike in the case of Ato1-GFP or Cit1-GFP (Figure 1C). However, we did find that the expression of another putative mRNA deadenylase, *NGL3*, was enriched in the colony center (Dataset S1). This prompted us to test for differential posttranscriptional mRNA regulation in colonies (inside versus outside), versus planktonic cells (stationary, which metabolically resemble colonies, and logarithmically growing cells). These comparisons allowed us to differentiate regulatory events that likely occur due to metabolic changes and nutrient starvation (these should be evident in the logarithmic versus stationary planktonic cultures), from those specific for colonies (which should only be evident in the planktonic versus colony comparisons). For these experiments, we chose several genes that showed altered gene expression on the outside or inside of colonies in our transcriptome analysis (Figure 4). To simultaneously confirm the differential expression identified by the array experiments, and to probe 3′UTR dynamics, we applied the semi-quantitative TVN-PAT and ePAT methods [17]. These methods report an invariant short poly(A)-tail or the full poly(A)-tail respectively. Both methods also detect alternative polyadenylation site usage. *ATO1* showed increased expression on the outside of the colony, and the mRNA in these cells showed an overall longer poly(A)-tail suggesting better translation (note longer smear of increasing amplicon size in ePAT reaction). *ATO1* is strongly expressed with alternate 3′UTRs in both 4-Day colony and planktonic stationary cultures, but expression is low in log phase cultures. However, a higher PCR cycle number revealed that the low level of *ATO1* that is expressed in logarithmic cultures has a significantly longer 3′UTR (see *ATO1* ↑cycles panel). The glycolytic enzyme *ENO1* is also clearly enriched on the outside of the colony whereas *NGL3*, *HSP30,* and the U6 spliceosomal RNA, *SNR6* are enriched in the cells representing the colony interior, confirming the array data.

To confirm the co-ordinate expression of regulated genes and nearby non-coding RNA as illustrated in Figure 3, we monitored the expression of SUT350 and *HPF1*, which likely share a bidirectional promoter. SUT350 was among the most enriched transcripts of the colony interior whereas *HPF1* is a cell wall mannoprotein expressed preferentially on the outside of the colony. *HPF1* is also expressed in log phase cultures. SUT350 is expressed as transcripts of different sizes depending on the metabolic state of cells, consistent with previous data suggesting heterogeneous transcription initiation and termination sites [33]. Cells on the outside of the colony and logarithmically growing cells express predominantly an intermediate-sized SUT350 transcript (see 2* in Figure 4). On the other hand, cells of the colony interior and stationary phase cultures express a distribution of transcripts, including very short and very long forms (see 1* and 3*) that were under-represented on the outside layer of the colony and in log cultures.

## Discussion

### Metabolism in the Yeast Colony

Our analysis of differential regulation of gene expression in the center (inside) and margin (outside) of a yeast colony revealed profound differences between the two subpopulations in regards to growth and metabolism. The outside cells displayed higher levels of genes required for ribosome biogenesis and expressed enzymes required for the fermentation of glucose, suggesting active fermentative growth. The inside cells appeared not to be growing actively: they did not display enrichment in ribosome biogenesis genes, and several functions which were enriched are a hallmark of nutrient deprivation, such as genes with functions in gluconeogenesis and those responding to nitrogen catabolite repression. As noted in the results, we observed that expression of both activators and repressors of gluconeogenesis and non-fermentative growth was present in the inside cells. For example the transcription factors *CAT8* (activator of gluconeogenesis) and *MIG1* (repressor of gluconeogenesis) were both expressed, as were the key enzyme of gluconeogenesis, the phosphoenolpyruvate carboxykinase *PCK1*, and three genes required for the turnover of another key gluconeogenesis enzyme, the fructose 1, 6 biphosphatase *FBP1* (Table 3). This could mean that longer term survival of the cells on the inside of the colony requires careful balancing of metabolic functions to ensure viability, but not exceed the capacity for growth. Of note, although generally the outside cells were expressing functions consistent with active growth, they also expressed genes which are hallmark of nutrient deprivation, most notably those required for fatty acid oxidation and acetyl-CoA synthesis (Table 1).

Mitochondrial functions were enriched in both subpopulations (Tables 1 and 3), but a closer inspection revealed that the cells residing in the inside of the colony expressed a larger proportion of genes required for the activity and assembly of the respiratory chain. The inside cells also expressed genes related to Fe-S cluster biogenesis and iron availability, which was not observed in the outside cell layers. The two key pathways requiring iron, Fe-S biogenesis and heme biosynthesis, both require mitochondrial function. These results indicate that the cells on the inside of the colony are attempting to maintain active mitochondrial function. Similar conclusions on differential metabolic regulation in the colony subpopulations were reached by a study published while this manuscript was in preparation. In that study, giant *S. cerevisiae* colonies were analyzed in the ammonium producing stage (i.e. in an analogous developmental phase as used in our study), and the inside cells were fractionated from the outside cells by size [34]. While the study by Cap et al also concluded that the outside cells grow actively and ferment glucose, while the inside cells maintain mitochondrial activity and non-fermentative metabolism, there were some differences in their results compared to ours. Cap et al did not note an enrichment of ergosterol biosynthesis genes on the outside of the colony (although some gene encoding ergosterol biosynthesis enzymes were up-regulated in the outside layers in their experiments), or Fe-S cluster biosynthesis and iron-related functions on the inside (again, some gene were shared between our experiments and Cap et al, such as *CAD1* and *NBP35*, but we found more pronounced differences) [34]. Compared to Cap et al, we observed a more modest enrichment of mitochondrial biogenesis in the inside of the colony, which in our case was confined to the respiratory chain and iron-sulfur cluster biogenesis genes, such as *SSQ1* and *ISU2*, while Cap et al reported enrichment of a large number of mitochondrial ribosomal proteins and chaperons (2012). These subtle differences could be due to somewhat different experimental set-ups and population isolation methods.

### Noncoding Transcription and Posttranscriptional Gene Regulation of Colony Development

Our results suggest that, in addition to the expression of protein-coding genes, noncoding transcription also shapes the transcriptome of the outside and inside colony layers. We found several CUTs and SUTs enriched in the two subpopulations of colony cells. Mapping these noncoding transcripts showed that often the genes in their neighborhood were also differentially regulated. As shown in Figure 3, several scenarios were observed, including expression from bidirectional promoters, the sense transcription of the noncoding transcript and the coding gene, and antisense transcription. At the present, it is not clear if or how the noncoding transcripts regulate the expression of the coding genes in the colony, but it is possible, and likely, that our observations reflect true regulatory relationships. An exciting question for the future is to test the noncoding-coding transcript pairs or trios in some cases, to understand this fascinating regulation.

Our data further indicates that posttranscriptional mRNA regulation contributes to gene regulation in the distinct metabolic and developmental state of cells growing in a colony. We observed differences in mRNA poly(A) tail length distribution and 3′ UTR usage between colony and planktonically grown cells for several genes which displayed altered expression levels in the colony (Figure 4). The stress protein *HSP30* for example, is expressed in all tested conditions and the presence of two distinct PCR amplicons indicates alternative 3′UTR usage. Cells on the inside of the colony, and stationary phase cells utilize predominantly the short 3′UTR isoform. Cells on the outside of the colony additionally express a longer 3′UTR isoform, which is the main form expressed in log phase cultures. Intriguingly, most transcripts show a smear of amplicons reflecting the distribution of poly(A)-tails of new and aging mRNA transcripts. The poly(A)-tail of *HSP30* in log phase cultures is universally long for both UTR isoforms. We speculate that this reflects an inactive/stored RNA population in these cells, whereas the mRNA within the colony and in stationary phase cells is actively translated and undergoes translation and age-associated deadenylation. These data, together with data showing differential adenylation site usage within the SUT350 transcriptional locus support our suggestion that the two populations within the yeast colony reflect distinct developmental states, opening a new field of study into the control of 3′ UTR dynamics in the development of colonies and other multicellular fungal structures.

### Parallels between *S. cerevisiae* Colonies and *Candida* Biofilms: Insight into How Heterogeneity of Growth and Metabolic States Shapes Biofilm Phenotypes

Several metabolic changes in yeast colonies that we observed in our transcriptome analysis have been previously reported to occur during biofilm growth of *C. albicans* and also *Candida parapsilosis* [7], [8], [9], [12]. These include expression of ribosome biogenesis and translation-related functions, glycolysis genes, genes required for ergosterol biosynthesis and fatty acid metabolism, amino acid metabolism and iron-related functions, as well as genes encoding cell wall functions. This is quite striking, as *C. albicans* biofilms are morphologically very different to *S. cerevisiae* colonies of lab strains. For example, unlike colonies of laboratory *S. cerevisiae* strains, *C. albicans* biofilms contain both yeast and filamentous cells and depend on adherence. Moreover, *C. albicans* biofilms are grown under very different conditions than what we used for our study. Using “*the awesome power of yeast genetics*” in the study of multicellular biofilms would be very beneficial, as pathogens such as *C. albicans* are genetically much less tractable then *S. cerevisiae*. In regards to lab strains, growth as a mat on semi-solid substrate of Sigma1278b was previously suggested to be a model for biofilms, mainly due to the requirement for adherence [36]. Our result, that growth of *S. cerevisiae* in a colony resembles *Candida* biofilms in regards to transcriptome changes, suggests that colony growth of laboratory strains, such as BY4741 that was used in our study, could be a good model for fungal biofilms. Therefore, the multitude of functional genomics tools in lab strains of *S. cerevisiae* could be explored for understanding biofilm formation in greater detail.

Our transcriptome profiling of the two subpopulations within a yeast colony provides insight into the heterogeneity of fungal multicellular structures, and the contributions of the different cell populations to the phenotypes associated with growth in communities. Most of the metabolic and growth reprogramming observed in both biofilms and colonies occurs in the outside colony layers (e.g. enrichment of genes with functions in ribosome biogenesis, glycolysis, ergosterol biosynthesis, fatty acid metabolism). Enrichment in amino acid metabolism genes is a hallmark of *C. albicans* biofilms, and is observable in both the outside and inside colony layers. A notable difference is in the expression of genes with functions in the sulfur assimilation pathway, which are strongly induced in *C. albicans* biofilms [7], [8], but not in a colony. However, some genes in this category were enriched in the inside colony layers, such as the adenylysulfate kinase *MET14* and the S-adenosylmethionine synthetase *SAM2* (Table 3). This result indicates that the changes in sulfate assymilation in biofilms could be due to the metabolic state of the cells in the center. The cell wall undergoes profound changes in a *C. albicans* biofilms compared to the planktonic state, for example the cell wall proteome is substantially remodeled (reviewed in [2], [3]). Another characteristic of *C. albicans* biofilms is production of extracellular matrix, which is important for biofilm cohesiveness and antifungal drug resistance [35], [37]. The extracellular matrix in *C. albicans* biofilms consists largely of ß 1,3 glucans, the main carbohydrate component of the fungal cell wall, and matrix production is linked to pathways that regulate cell wall functions [35], [38]. Our data suggests that the different colony environments to which the outside and inside cells are exposed impinge in a distinct manner on cell wall functions. The cell wall proteome is remodeled in the outside cells, and also the expression of the *FKS1* glucan synthase is enriched, while the cells in the center of the colony express cell wall degrading enzymes such as glucanases and chitinases. It is interesting that the homolog of the only *C. albicans* gene expression regulator of matrix production reported so far, the zinc-responsive transcription factor Zap1 [39], is also enriched on the inside of *S. cerevisiae* colonies (Table 3). An exciting area for the future is to decipher how this differential regulation of cell wall functions in the different subpopulation links into the pathways controlling cell wall remodeling and matrix production in fungal biofilms. An important contribution in this context could be from changes to mitochondrial activity, which are observed within the colony. The biofilm environment is likely hypoxic, and hypoxia has been suggested to be responsible for several biofilm characteristics, such as activation of genes required for ergosterol biosynthesis and glycolysis [9], [40]. Hypoxia would impact on mitochondrial function. Mitochondrial function is important for cell wall integrity in several fungal species, including *S. cerevisiae*, *C. albicans* and *C. glabrata* [41], [42], [43], [44], [45]; reviewed in [46]). In some cases, links between mitochondrial activity and cell wall ß-glucans have been reported [41], [43]. It could therefore be envisaged that changes to mitochondrial function in a fungal community of cells could be contributing to cell wall restructuring and production of the extracellular matrix.

## Supporting Information

Dataset S1 CIT 1 sorted by fold change.
(XLS)
